# Prevalence of Stroke Risk Factors and Their Distribution Based on Stroke Subtypes in Gorgan: A Retrospective Hospital-Based Study—2015-2016

**DOI:** 10.1155/2018/2709654

**Published:** 2018-07-26

**Authors:** Mahdi Habibi-koolaee, Leila Shahmoradi, Sharareh R. Niakan Kalhori, Hossein Ghannadan, Erfan Younesi

**Affiliations:** ^1^Clinical Research Development Unit (CRDU), Sayad Shirazi Hospital, Golestan University of Medical Sciences, Gorgan, Iran; ^2^Halal Research Center of IRI, FDA, Tehran, Iran; ^3^Health Information Management Department, School of Allied Medical Sciences, Tehran University of Medical Sciences, Tehran, Iran; ^4^Department of Neurology, School of Medicine, Golestan University of Medical Sciences, Gorgan, Iran; ^5^Information Technology for Translational Medicine, L-4362 Esch-sur-Alzette, Luxembourg, Luxembourg

## Abstract

**Background:**

Stroke is a leading cause of death and disability worldwide. According to the Iranian Ministry of Medical Health and Education, out of 100,000 stroke incidents in the country, 25,000 lead to death. Thus, identifying risk factors of stroke can help healthcare providers to establish prevention strategies. This study was conducted to investigate the prevalence of stroke risk factors and their distribution based on stroke subtypes in Sayad Shirazi Hospital, Gorgan, Northeastern Iran.

**Material and Methods:**

A retrospective hospital-based study was conducted at Sayad Shirazi Hospital in Gorgan, the only referral university hospital for stroke patients in Gorgan city. All medical records with a diagnosis of stroke were identified based on the International Classification of Diseases, Revision 10, from August 23, 2015, to August 22, 2016. A valid and reliable data gathering form was used to capture data about demographics, diagnostics, lifestyle, risk factors, and medical history.

**Results:**

Out of 375 cases, two-thirds were marked with ischemic stroke with mean ages (standard deviation) of 66.4 (14.2) for men and 64.6 (14.2) for women. The relationship between stroke subtypes and age groups (P=0.008) and hospital outcome (P=0.0001) was significant. Multiple regression analysis showed that hypertension (Exp. (B) =1.755, P=0.037), diabetes mellitus (Exp. (B) =0.532, P=0.021), and dyslipidemia (Exp. (B) =2.325, P=0.004) significantly increased the risk of ischemic stroke.

**Conclusion:**

Overall, hypertension, diabetes mellitus, and dyslipidemia were the major risk factors of stroke in Gorgan. Establishment of stroke registry (population- or hospital-based) for the province is recommended.

## 1. Introduction

Stroke is the second leading cause of death, accounting for 11.13 % of total deaths, and the main cause of disability worldwide. The major type of stroke is ischemic, which occurs in about 87% of all stroke cases [1]. According to Global Burden of Disease (GBD) study in 2010, more than 11 million ischemic strokes occurred while 63% of them were in low- and middle-income countries. Also, near 3 million deaths occurred due to ischemic stroke [2]. About 13% of stroke is of a hemorrhagic type [1]. According to GBD study in 2010, there were about 5.3 million hemorrhagic stroke cases, out of which about 80% occurred in low- and middle-income countries. Over 3 million deaths occurred from hemorrhagic stroke [3]. The range of incident rates of stroke in Iran varies [4]. According to the Mashhad Stroke Incidence Study, conducted during 2006-2007, the age-adjusted incidence rate of stroke was 203 per 100,000 individuals per year (95 % CI: 175-231) and this rate could be divided based on the stroke subtype into 113 (95% CI: 142-192), 26 (95 % CI 16-36), and 4 (95 %CI 0-8) per 100000 per year for ischemic, intracerebral hemorrhage, and subarachnoid hemorrhage stroke, respectively [5].

The incidence rate of stroke in middle east countries is variable [6] and it is progressively becoming a main problem in the region [7]. This rate in Iranian population is increasing dramatically [6, 8, 9]. For example, in Isfahan Cohort Study (ICS) [9], which prospectively followed up 6504 individuals (32893 person-years of follow-up) between 2003 and 2010, 91 new stroke cases (43 men and 48 women) were confirmed. The age-adjusted incidence rate was 260 (95% CI: 179-341), 295 (95% CI: 205-384), and 280 (95% CI: 219-341) per 100,000 person-years for men, women, and total, respectively. The stroke mortality rate was 49 (95% CI: 13-85), 68 (95% CI: 20-116), and 59 (95% CI: 29-89) per 100,000 person-years, for men, women, and total, respectively. There were significant differences in stroke rates among women (P value= 0.020), when compared to the incidence rates nine years later. The authors concluded that the urgent consideration by health policy makers is essential for this high incident rate of all cardiovascular diseases, especially stroke and its mortality.

Stroke has different risk factors, which can be grouped into modifiable and nonmodifiable risk factors. Major risk factors for stroke include age, history of cerebrovascular event, smoking, alcohol consumption, physical inactivity, hypertension, dyslipidemia, diabetes mellitus, cardiovascular diseases, obesity, metabolic syndrome, diet, nutrition, and genetic risk factors [10–12]. In Iran, many hospital-based studies have investigated the stroke risk factors. Hypertension, diabetes mellitus, ischemic heart diseases, male gender, and age >=65 years are main reported stroke risk factors in Iran [7, 13]. To our knowledge, there is only one study reporting on stroke risk factors in Gorgan, the capital of Golestan province, northeastern Iran, but it lacks details on distribution of risk factors over the stroke subtypes [14]. According to this study, which was accomplished in 2001, the major risk factors were hypertension (64%), hyperglycemia (29.3 %), cigarette smoking (22.7 %), and heart diseases (22%).

The aim of the present study was to investigate the stroke subtypes and risk factors in patients admitted to Sayad Shirazi Hospital affiliated to Golestan University of Medical Sciences (GoUMS) in Gorgan. The Sayad Shirazi Hospital is the only referral university hospital for stroke patients in Gorgan city.

## 2. Materials and Methods

Medical records of all patients who were admitted to the Sayad Shirazi Hospital, affiliated to GoUMS, between August 23, 2015, and August 22, 2016, were manually screened for a confirmed discharge diagnosis of stroke (International Classification of Diseases, Revision 10 (ICD 10) codes in the categories of I60, I61, I62, I63, and I64). Diagnosis was mainly based on physician's opinion, clinical features, and Magnetic Resonance Imaging (MRI) or Computed Tomography (CT scan) reports. Medical records with a diagnosis of unspecified stroke (code I64 of ICD 10) were rechecked with a specialist coder to assign a correct code based on MRI or CT scan reports or after consulting with the attending physician or a neurologist.

A valid and reliable data collection form was used to capture data contained in the medical records. In this form, the following criteria and clinical parameters were considered: patient demographic data (age, gender, ethnicity, and residence area), diagnostic data, type of stroke (hemorrhagic or ischemic), history of diseases (such as hypertension, ischemic heart disease, nonischemic heart disease, diabetes, hyperlipidemia, previous stroke, or Transient Ischemic Attack), and lifestyle data (such as cigarette smoking and opium addict).

The chi-square test and Mann–Whitney U test were used to analyze the categorical and continuous data, respectively. The relationship between risk factors and stroke subtypes were examined by logistic regression and chi-square test. Statistical significant was P value<0.05. All statistical analyses were performed by SPSS software, version 20 (IBM SPSS Statistics).

This study was approved by the ethical committee and the institutional review boards of the GoUMS (code: IR.GOUMS.REC.1395.205). The permission to collect data from medical records was provided by the Sayad Shirazi Hospital administration.

## 3. Results

In the study period, the medical records of 25422 patients were surveyed for stroke diagnostic code based on ICD 10. Our screening protocol identified 415 stroke records, out of which 40 cases (9.6 %) were unspecified stroke. Of 375 specified stroke cases, 70.7% described an ischemic incidence and 29.3 % reported a hemorrhagic incidence. Analysis of demographic attributes over this dataset showed that 218 (58.1%) men and 157 (41.9%) women with mean ages (standard deviation) of 66.4 (14.2) and 64.6 (14.2), respectively, were admitted with stroke diagnosis, irrespective of stroke type. Further analysis indicated that both ischemic and hemorrhagic stroke subtypes had high prevalence in age >=70 years (Table 1). The relationship between age group and stroke subtype was significant (P<0.05) but there was no significant association between ethnicity and stroke subtypes (P value =0.335). The majority of ethnicity was Fars.

Chi-square test showed that the hospital outcome differs based on stroke subtypes (p<0.05). Analysis of the mortality rate in this population based on the stroke subtype indicated that the mortality rate in patients hit by hemorrhagic stroke is higher than those with ischemic attack. Furthermore, there was no significant difference between stroke subtypes, on one hand, and gender and residence, on the other hand (Table 2).

Analysis of possible relations between comorbid risk factors and stroke subtypes using chi-square test showed that, compared to other comorbid risk factors, diabetes mellitus (P value=0.003) and dyslipidemia (P value=0.001) were significantly prevalent among ischemic and hemorrhagic stroke patients. There was no significant difference between ischemic and hemorrhagic stroke with regard to other risk factors [Table 3].

The regression model of comorbid risk factors indicated that the risk of ischemic stroke in patients with dyslipidemia and hypertension is 2.325 and 1.755 higher than patients suffering from hemorrhagic stroke, respectively. Also, the risk of ischemic stroke is 47% higher than hemorrhagic stroke in diabetic patients [Table 4].

## 4. Discussion

According to the global stroke belt report in 2015, Iran ranks 187 among other countries, representing one of the highest stroke incidence rates in the world [15]. The Iranian Stroke Society reports that this rate continues to increase so that 300 stroke patients are admitted to hospitals every day and the average age of stroke onset in Iran is getting 10 years younger than the global average age (https://goo.gl/pEDZZW). This alarming situation calls for identification of stroke risk factors among the Iranian patient population so that prevention strategies can be defined and risk factors can be managed properly.

The existing risk factor studies on the Iranian stroke patients are relatively old [4, 16] but our results are in line with their reports. For instance, Ahangar et al. (2005) reported that the incidence of stroke among female patients in Babol was relatively higher than male patients and the average age of stroke incidence was 68 [17] whereas our study in Gorgan not only shows a reverse gender distribution but also confirms the fact that the average age of stroke incidence among stroke patients is declining to younger ages. Another finding in this patient population was the higher mortality rate among patients hit by the hemorrhagic stroke, which indicates that this group of patients is exposed to fatal aneurysmal ruptures in the brain and such incidences might be prevented by early diagnosis and screening for unruptured aneurysms [18]. Moreover, the observed higher rates of ischemic stroke incidence suggests that ischemic stroke patients have a great exposure to modifiable risk factors whose control through lifestyle modifications can prevent a large proportion of such incidences [19]. Interestingly, those patients who were resident in urban districts were more than 1.5 times at higher risk of developing stroke compared with residents of rural areas, which can be probably attributed to the unhealthy lifestyle practices with regard to lack of physical activity and unhealthy diet [20].

In general, a strong relationship between some risk factors (such as hypertension, diabetes mellitus, dyslipidemia, smoking, and age) and the incidence of stroke has been reported in the literature [21]. It is now established that hypertension is a major risk factor for stroke, both ischemic and hemorrhagic [22]. In our study, although the most frequent risk factor in both ischemic (30.6 %) and hemorrhagic (26.3 %) stroke subtypes is hypertension, the chi-square test showed that these results were not statistically significant (P>0.05). Instead, the regression model of all risk factors showed that the prevalence of hypertension in ischemic stroke patients is 1.755 times higher than hemorrhagic stroke patients. According to Malekzadeh et al. (2013), the WHO age-standardized prevalence of hypertension in Golestan province was 41.8% (95%CI: 38.3%–45.2%) [23]. In the study of Hosaini and colleague (2001) [14], 64% of stroke patients in Gorgan—regardless of the stroke type—had hypertension, which is similar to our study (66.9 %). Recently, a prospective study of 50,000 adults in the Golestan Cohort by Sepanlou and colleagues (2016) [24] showed a positive association between hypertension and stroke mortality: they found that the stroke mortality rate was 147.1 (95%CI: 133.9-160.1) per 100,000 person-years, although they did not consider stroke subtypes. With regard to the stroke subtype, our finding that prevalence of hypertension was higher among ischemic stroke patients is consistent with findings of Bilic et al. (2009) [25] and Zhang et al. (2011) [26] who reported similar results in Croatian and Chinese stroke populations, respectively. The role of hypertension in pathogenesis of stroke has been well documented in other studies, which include the initiation of vasculopathy, promotion of microatheroma, lipohyalinosis, and atherosclerotic diseases, and blood-brain barrier disruption [27]. According to a meta-analysis study of clinical trials by Law et al. (2009), the incidence rate of stroke was dropped by 41% when both systolic and diastolic blood pressure were reduced [28]. It seems that antihypertensive therapy and lifestyle modification can reduce the risk of stroke and its recurrence. Since hypertension develops by aging, lowering hypertension risk profile through lifestyle changes starting in middle age can prevent cardiovascular events in older ages [29]. Indeed, the CARDIA (Coronary Artery Risk Development in Young Adults) study demonstrates that, beside physical activity, diet is the most prevalent risk factor for cardiovascular diseases [30]. This study recommends that clinicians should adopt dietary approaches to stop hypertension during young adulthood to middle age as healthier diet significantly contributes to lower risk of developing hypertension and consequently cardiovascular events such as stroke in the elderly.

Another important finding in our study was positive and significant association between diabetes mellitus and incidence of stroke, particularly the ischemic subtype. According to our finding, 36% of all stroke patients were diabetic and this is in agreement with Hosaini and colleagues' [14] findings, which showed 29.3% of all stroke patients had hyperglycemia. The prevalence of diabetes in ischemic stroke patients was 47% higher than that in patients with hemorrhagic stroke. Based on the Golestan Cohort Study between 2004 and 2007, it appeared that a crude prevalence of diabetes mellitus in Golestan province was about 6.9 % (95%CI: 6.7-7.1), which was positively associated with obesity at all ages [31]. Diabetes causes several metabolic and pathologic changes that lead to stroke, including arterial stiffness, systematic inflammation, endothelial dysfunction, and heart failure [32, 33]. By these changes, stroke in a diabetic patient increases hospital mortality [34] as reflected in our results (odds ratio: 7.341 with 95% CI: 3.637-14.817). Controlling diabetes can prevent both primary and secondary stroke and may decrease the mortality as well. Clinical studies suggest that improvements in nutrition and dietary pattern, beside weight management, in diabetic patients lower cardiovascular disease incidence significantly [35]. Thus, dietary components and nutritional patterns should be incorporated into any prevention strategy at the national level [36].

In the current study, we also found that dyslipidemia was strongly associated with the incidence of total stroke (P<0.05), which was consistent with previous findings reported by Tziomalos et al. [37] and Sarti et al. [38]. As expected, prevalence of dyslipidemia in ischemic stroke patients was almost double the hemorrhagic stroke cases. Atherosclerosis is the major mechanism of dyslipidemia, which leads to stroke [39]. It seems that reducing lipid profile can reduce atherosclerotic plaques, which results in the decreased risk of stroke. Very recently, the Strong Heart Study (2017) revealed that low density lipoprotein cholesterol levels equal to or greater than 130 mg/dl confer higher risk of ischemic stroke, but interestingly individuals with high triglycerides and low high density lipoprotein levels who were also suffering from diabetes showed more than 2 times higher incidence of ischemic stroke [40]. As previously reported, there is a significant association between dyslipidemia and diabetes. This observation implies that coincidence of two risk factors, namely, diabetes and dyslipidemia, already doubles the risk of ischemic stroke incidence and such at-risk population should be given priority in prevention programs.

This study confirms that age >=70 is associated with stroke risk (p<0.05), which was similar to other studies in Iranian population [7]. However, the observation that more than half of all stroke cases were below 69 years (57%) and nearly half of hemorrhagic cases (47.4%) were younger than 59 years confirms the trend of excessive stroke incidence among Iranians at younger ages [5]. This trend can be attributed to a shift in prevalence of vascular risk factors towards younger adults; a meta-analysis of ischemic stroke data from three large studies showed a sharp increase in the prevalence of hypertension followed by hypercholesterolaemia over the age of 35 years and interestingly a striking increase in the number of risk factors per patient over 35 years of age [41].

## 5. Conclusion

The present study was designed based on hospital medical records to determine the prevalence of stroke subtypes and their relations to risk factors in Gorgan, Northeastern Iran. Since analysis of medical record data for evidence-based medicine becomes an indispensable part of prevention and treatment strategies, the quality of these records in hospitals is of high importance. In the studied population of stroke patients at Gorgan, the prevalence of stroke incidence and rates of mortality were significantly related to known comorbid risk factors such as hypertension, diabetes, and dyslipidemia but no significant association was found with ethnicity. Moreover, a relatively high prevalence of stroke incidence in younger age groups was alarming and in agreement with recent trends. These findings may be indicative of a stronger role for environmental risk factors than genetic ones in incidence of stroke subtypes in the studied population.

## Limitations and Recommendation

The current study was limited by the quality of medical records documentation due to incompleteness of some parameters such as weight, height, exercise, and occupation. In the case of accuracy, diagnoses were not documented accurately by the physician. To correct this, we had to consider all reports and sometimes refer to attending physician, particularly for medical records with a diagnosis of unspecified stroke type (ICD 10 code of I64). An implication of these findings is that these risk factors should be taken into account for decision making at GoUMS when developing preventive strategies. The observation that the risk of ischemic stroke in patients with hypertension, dyslipidemia, and diabetes mellitus was higher than hemorrhagic stroke calls for a prevention plan at the regional level with focus on major lifestyle factors, namely, diet, exercise, smoking, and body mass index. Control and treatment of hypertension and dyslipidemia through lifestyle modifications and medical management should be given high priority in the studied patient population. We foresee a need for implementation of a cohort- or population-based study of stroke in the region. There is, therefore, a definite need for the establishment of hospital- or population-based stroke registry at regional or national levels.

## Figures and Tables

**Table 1:** Distribution of age and ethnicity and their association with stroke subtypes.

	Hemorrhagic stroke	Ischemic stroke	Total	p value
Age group (N., %)				
<40	5 (4.5)	7 (2.6)	12 (3.2)	0.008
40-49	14 (12.7)	19 (7.2)	33 (8.8)	
50-59	31 (28.2)	43 (16.2)	74 (19.7)	
60-69	19 (17.3)	76 (28.7)	95 (25.3)	
>=70	41 (37.3)	120 (45.3)	161 (43)	
Mean ±SD	62.93±15.68	67.31±13.49		0.011
Ethnicity (N., %)				
Fars	83 (27.8)	216 (72.2)	299 (79.7)	0.335
Turkmen	24 (36.9)	41 (63.1)	65 (17.3)	
Baluch/ Sistani	3 (27.3)	8 (72.7)	11 (3)	

**Table 2:** Distribution of gender, residence, and discharge status as well as their association with stroke subtypes.

	Hemorrhagic stroke	Ischemic stroke	Odds Ratio	95% CI	P value
Gender					
Male	63 (57.3)	155 (58.5)	0.951	0.607-1.492	0.828
Female	47 (42.7)	110 (41.5)			
Residence					
urban	69 (62.7)	168 (63.4)	0.972	0.613-1.540	0.903
rural	41 (37.3)	97 (36.6)			
Discharge status (hospital outcome)					
Death	29 (33.7)	14 (6.5)	7.341	3.637-14.817	0.0001
Live	57 (66.3)	202 (93.5)			

**Table 3:** Prevalence of comorbid risk factors of stroke.

	Hemorrhagic stroke	Ischemic stroke	Total	Odds Ratio	95 % CI	P value
Hypertension	77 (30.6)	174 (26.3)	251 (66.9)	0.819	0.507-1.325	0.416
Diabetes mellitus	27 (10.7)	108 (16.3)	135 (36)	2.115	1.284-3.482	0.003
Ischemic heart disease	20 (7.9)	57 (8.6)	77 (20.5)	1.233	0.700-2.172	0.468
Dyslipidemia	22 (8.7)	61 (15.4)	83 (22.1)	2.503	1.475-4.247	0.001
Transient ischemic attack	4 (1.6)	6 (0.9)	10 (2.7)	0.614	0.170-2.219	0.488
Other heart disease	11 (4.4)	15 (2.3)	26 (6.9)	0.540	0.240-1.216	0.132
Alzheimer disease	1 (0.4)	2 (0.3)	3 (0.8)	0.829	0.074-9.236	1
Anemia	1 (0.4)	3 (0.5)	4 (1.1)	1.248	0.128-12.131	1
Asthma	1 (0.4)	3 (0.5)	4 (1.1)	1.248	0.128-12.131	1
Chronic kidney disease	3 (1.2)	5 (0.8)	8 (2.1)	0.686	0.161-2.921	0.697
Old CVA	32 (12.7)	81 (12.2)	113 (30.1)	1.073	0.659-1.747	0.777
History of Cancer	2 (0.8)	7 (1.1)	9 (2.4)	1.465	0.300-4.166	1
Hypothyroidism	1 (0.4)	6 (0.9)	7 (1.9)	2.525	0.300-21.223	0.679
Kidney stone	2 (0.8)	9 (1.4)	11 (2.9)	1.898	0.404-8.932	0.520
Parkinson disease	1 (0.4)	1 (0.2)	2 (0.5)	0.413	0.026-6.660	0.501
History of surgery	4 (1.6)	11 (1.7)	15 (4)	1.148	0.357-3.685	1
Seizure	1 (0.4)	3 (0.5)	4 (1.1)	1.248	0.128-12.131	1
History of trauma	3 (1.2)	1 (0.2)	4 (1.1)	0.135	0.014-1.313	0.078
Hepatitis	3 (1.2)	1 (0.2)	4 (1.1)	0.135	0.014-1.313	0.078
Family history of hypertension	4 (1.6)	2 (0.3)	6 (1.6)	0.202	0.036-1.117	0.064
Family history of diabetes mellitus	2 (0.8)	1 (0.2)	3 (0.8)	0.205	0.018-2.279	0.207
Smoking	12 (4.8)	18 (2.7)	30 (8)	0.595	0.276-1.282	0.181
Opium	18 (7.1)	46 (6.9)	64 (17.1)	1.074	0.591-1.950	0.816

**Table 4:** Logistic regression analysis of risk factor in stroke.

	B	SE	Wald	df	Sig.	Exp. (B)
Dyslipidemia	0.844	0.290	8.434	1	0.004	2.325
Hypertension	0.562	0.269	4.355	1	0.037	1.755
Diabetes mellitus	-0.631	0.274	5.292	1	0.021	0.532

B: coefficient for the constant; SE: standard error; df: degrees of freedom; Sig.: significant; Exp. (B): exponentiation of the B coefficient.

## Data Availability

The data used to support the findings of this study are available from the corresponding author upon request.
